# Stress Analysis of Occlusal Forces in Canine Teeth and Their Role in the Development of Non-Carious Cervical Lesions: Abfraction

**DOI:** 10.1155/2012/234845

**Published:** 2012-07-30

**Authors:** Shihab A. Romeed, Raheel Malik, Stephen M. Dunne

**Affiliations:** ^1^Department of Restorative Dentistry, King's College London Dental Institute, Denmark Hill Campus, Caldecot Road, London SE5 9RW, UK; ^2^Department of Restorative Dentistry, Guy's Hospital, Tower Wing, Great Maze Pond, London SE1 9RT, UK

## Abstract

Non-carious cervical tooth lesions for many decades were attributed to the effects of abrasion and erosion mainly through toothbrush trauma, abrasive toothpaste, and erosive acids. However, though the above may be involved, more recently a biomechanical theory for the formation of these lesions has arisen, and the term abfraction was coined. The aim of this study was to investigate the biomechanics of abfraction lesions in upper canine teeth under axial and lateral loading conditions using a three-dimensional finite element analysis. An extracted human upper canine tooth was scanned by **μ**CT machine (Skyscan, Belgium). These **μ**CT scans were segmented, reconstructed, and meshed using ScanIP (Simpleware, Exeter, UK) to create a three-dimensional finite element model. A 100 N load was applied axially at the incisal edge and laterally at 45° midpalatally to the long axis of the canine tooth. Separately, 200 N axial and non-axial loads were applied simultaneously to the tooth. It was found that stresses were concentrated at the CEJ in all scenarios. Lateral loading produced maximum stresses greater than axial loading, and pulp tissues, however, experienced minimum levels of stresses. This study has contributed towards the understanding of the aetiology of non-carious cervical lesions which is a key in their clinical management.

## 1. Introduction

Abfraction has been defined as microstructural loss of dental tissues caused by biomechanical loading which leads to stress concentration in the cervical region of teeth which, in turn, leads to loss of tooth structure [1, 2]. Its clinical appearance has been described in the literature as early as 1930's [3], but its association with loading became apparent much later when Lee and Eakle hypothesized the role of occlusal loading in formation of cervical lesions [4]. There are three main types of stresses that are placed on teeth during mastication and parafunction: compressive, shear, and tensile stresses [4]. It seems that the role of tensile stresses in the aetiology of cervical lesions started to become more evident after finite element analysis research had indicated that that these stresses were concentrated in the cervical region of teeth. Consequently, the concentration of tensile stresses was linked to the cause of cervical lesions (abfraction) [5–7]. Occlusal forces generated during oral functions, parafunction, and premature contacts give rise to significant tensile stresses in the cervical enamel [8, 9]. Chen et al. indicated that there is proportional linear relational between occlusal forces and induced stresses both buccally and lingually [8].

A number of theories were put forward to justify how stresses at the buccocervical level result in lesions; enamel at the cervical region is known to be of poorer quality as thinner layers of enamel prisms are present in the cervical areas [10]. Therefore, it is not tough enough to withstand tensile stresses, which are usually concentrated at the tooth fulcrum found near the cervical region. In addition to that, enamel responds differently from dentine to forces causing differential flexure, which results in weakening, formation of microcracks. Once the load applied and stresses produced exceed the theoretical yield stress of enamel, the abfraction lesion is formed appearing as a wedge-shaped cervical lesion. Other theories suggest that erosive acids may also play a role, by weakening the structure of enamel and dentine in the buccocervical area, thus making the area more susceptible to fracture, aiding in the formation of the abfraction lesion.

## 2. Objectives

The purpose of this study was to investigate by means of three-dimensional finite element analysis (3D-FEA) the biomechanics of abfraction lesions in upper canine teeth under different loading conditions.

## 3. Materials and Methods

**Figure 1:**
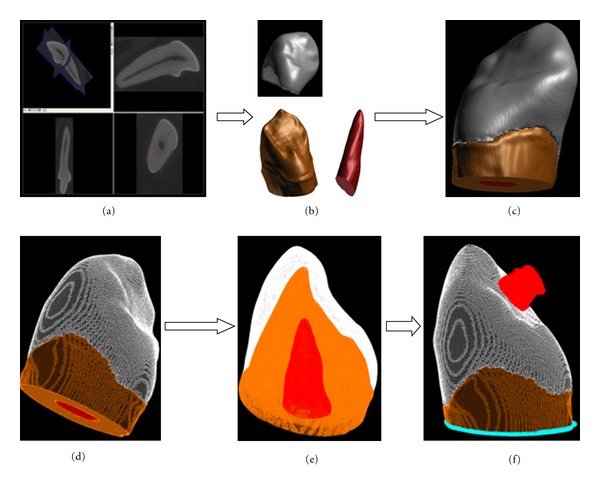
micro-CT imaging, modeling, and developing FE model of a canine tooth; (a) section of micro-CT; (b) segmentation and 3D reconstruction of tooth tissues; (c) 3D model of canine tooth; (d) FE mesh of 3D model; (e) assignment of different materials properties; (f) boundary conditions and lateral loading on 3D-FE model.

A human upper canine tooth, which was extracted for orthodontic reasons, scanned in a microcomputed-tomography scanner  (*μ*CT) (Skyscan, Kontich, Belgium). The resolution (voxel size) of this scan was 14 × 14 × 14 *μ*. All scans were imported by ScanIP (Simpleware, Exeter, UK) as stack of images for masking, segmentation, and three-dimensional (3D) reconstructions. Depending on pixel density three masks were generated representing enamel, dentine, and pulp tissues. Following 3D reconstruction and optimisation of the geometry surfaces, finite element (FE) mesh was generated on the 3D model using ScanFE (Simpleware, Exeter, UK). The FE model comprises 213220 nodes and 562998 tetrahedral elements (Figure 1). Subsequently, the FE model was imported by Patran (MScsoftawre, Santa Ana, CA, USA) for pre- and post processing. All materials were considered to be isotropic, homogenous and linear elastic. The details of the mechanical properties of all materials included in the FE model are included in Table 1. Boundary conditions were set out as the whole model was constrained at its base in all directions (*X* = 0, *Y* = 0, *Z* = 0, *XY* = 0, *XZ* = 0, and  *YZ* = 0). Axial loading 100 N was applied separately at the tip of incisal edge and laterally mid of the palatal surface at 45°  to the long axis of the canine tooth. In a different loading scenario, the canine tooth was subjected to 200 N axial and non-axial loading simultaneously in both directions (Figure 1). Nastran (MScsoftware, Santa Ana, CA, USA) was used to perform all FE solutions and calculate stresses within the canine tooth and its displacement under three different loading scenarios. Comparative FE stress analyses were carried out to identify maximum von Mises stresses (the equivalent stress of principal stresses in *X*, *Y*, *Z* directions) and their distribution and:

(1) von  Mises  stress  =12[(σ1−σ2)2+(σ2−σ3)2+(σ3−σ1)2],

where *σ*1, *σ*2, and *σ*3 are principal stresses and *σ*1 > *σ*2 > *σ*3.

## 4. Results

**Figure 2:**
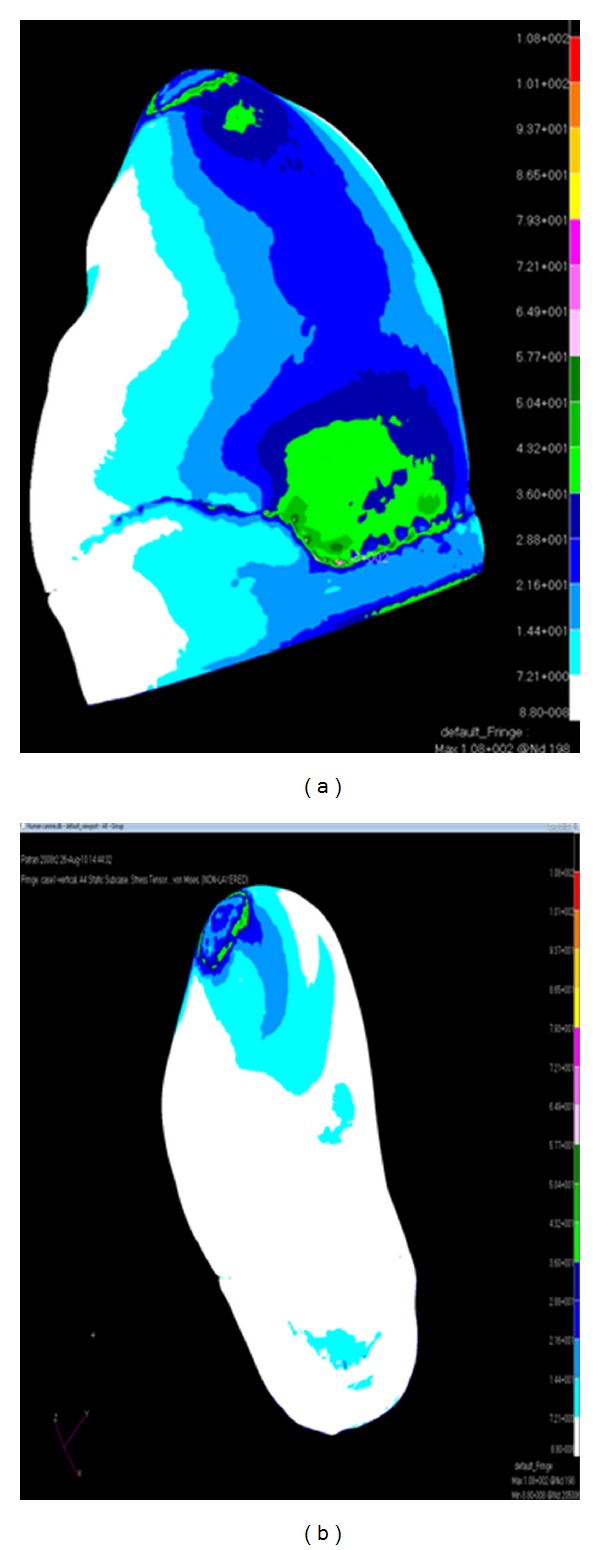
von Mises stress distribution caused by 100 N vertical load ((a) buccal view and (b) lingual view).

**Figure 3:**
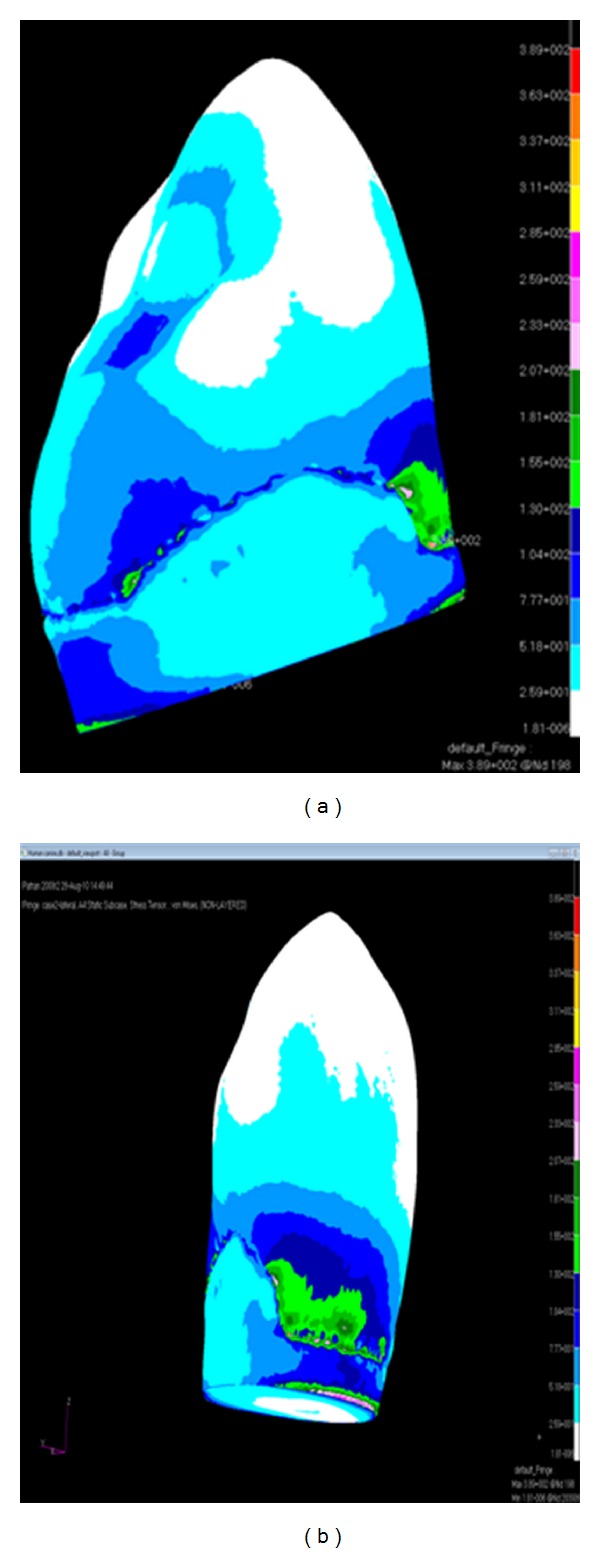
von Mises stress distribution caused by 100 N lateral loading ((a) lateral view and (b) buccal view).

**Figure 4:**
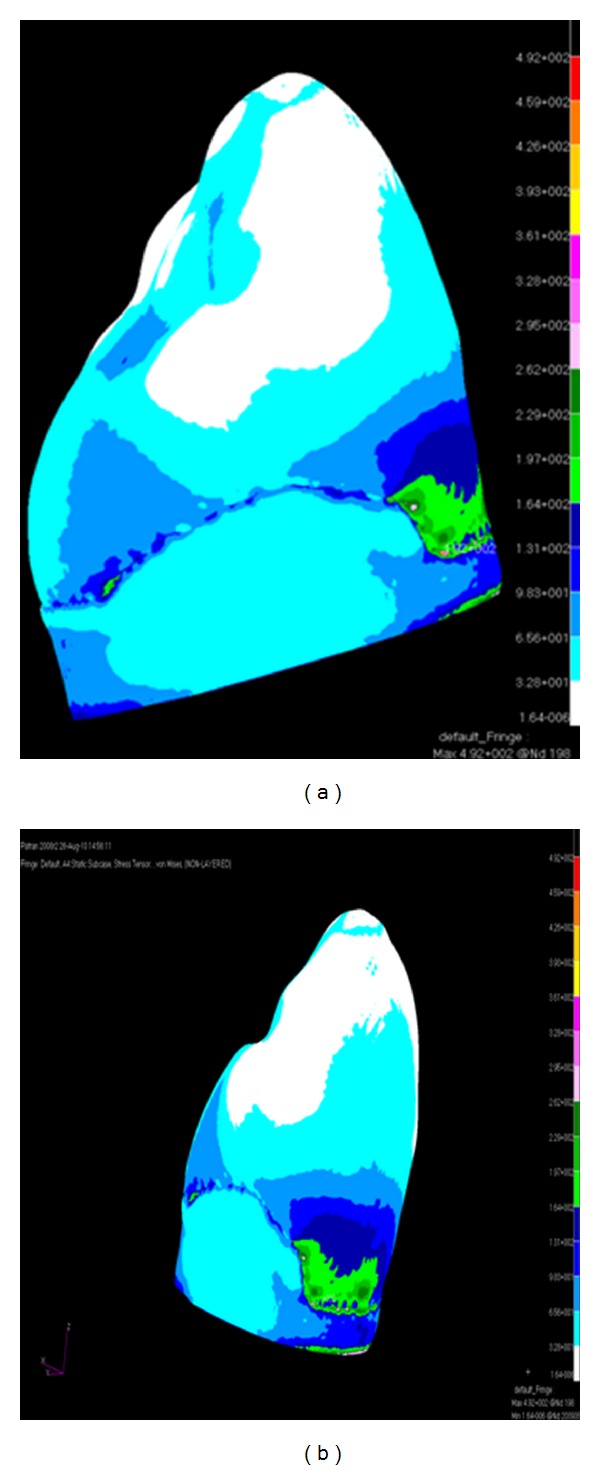
von Mises stress distribution caused by 200 N vertical and lateral load ((a) lateral view and (b) buccal view).

**Figure 5:**
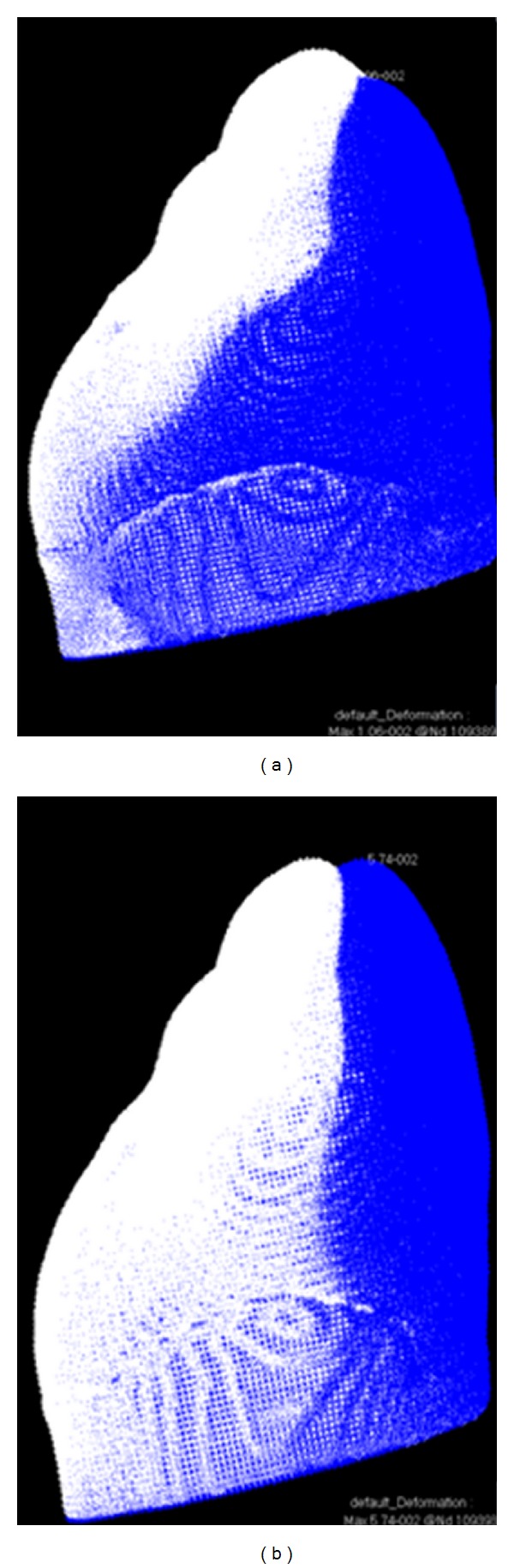
Enamel's displacement caused by 100 N vertical loading (a) and lateral loading (b).

**Figure 6:**
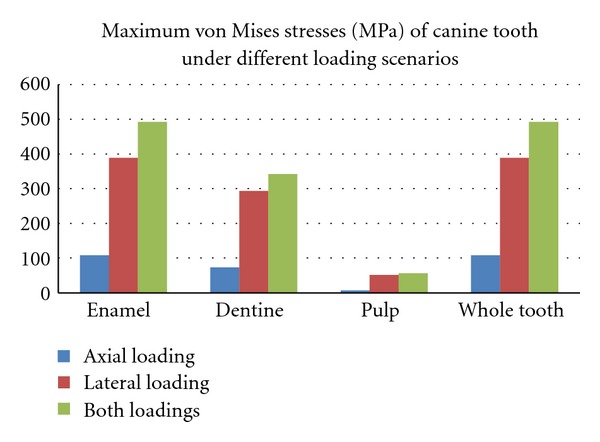
Maximum von Mises stresses (MPa) in tooth tissues under different loading scenarios.

When the canine tooth was subjected to 100 N axial loading, maximum von Mises stresses generated in enamel (108 MPa) was higher than the dentine (73 MPa) (Figure 2). The change of the force angulation (45° to the long axis) increased the level of maximum von Mises stresses drastically, enamel suffered 3 times greater increase (389 MPa) in stress concentration compared with dentine (Figure 3). However, the location of maximum stresses was similar, located at the buccal cervical region close to the cementoenamel junction (CEJ). The maximum von Mises stress was much greater in enamel (492 MPa) at the buccal CEJ, when load was applied in both directions simultaneously (Figure 4). Dentine behaved similarly as stresses concentrated at a similar location, although the maximum value was reduced by 25% (342 MPa) (Table 2). Displacement of tooth structure in both enamel and dentine was about 55 micron under lateral loading five times higher than axial loading (Table 2 and Figures 5 and 6).

## 5. Discussion

Early detection and diagnosis of abfraction lesions are important so that lesions can be stabilized and prevented from progressing. Abfraction might take different forms, that is, hairline cracks, striations, saucer-shaped lesion, or crescent-shaped lesion [11]. Abfraction may also occur along side other forms of tooth surface loss and these also need to be diagnosed in order to achieve appropriate treatment planning.

Taking into account the size of the lesion, clinicians may decide to leave and monitor the lesion or actively treat it with or without any occlusal adjustment. However, it is evidence-based that such lesions should be restored to prevent further progression, protect against pulp exposure and tooth fracture, and improve aesthetics [12, 13]. Further studies have shown that any restoration placed at this site is at an increased risk of failure due to continued presence of tensile stresses [14], therefore treatment should only be instigated in the aforesaid situations where leaving a lesion to progress might have irreversible squeelae [15].

Currently, there is a lack of evidence to support the trend that occlusal adjustment would alter the forces applied to tooth structure especially in the upper canine tooth which usually leads lateral excursions in canine-guided occlusion. Therefore, the disparity of stresses between enamel and dentine indicates that the aetiology of abfraction is multifactorial including occlusal forces [16].

This study has employed 3D-FEA to investigate the influence of axial and non-axial occlusal loading in canine teeth on the development of abfraction lesions. It was aimed to get an insight into the development of stress distribution within tooth tissues and along the enamel dentine interface under different loading conditions. Although some 3D-FEA studies have indicated the role of periodontal ligament (PDL) as an important factor in the pattern of stress distribution within dental tissues [17], PDL has not been included in the 3D model of this study due to the difficulty in getting uniform thickness of PDL around the canine root based on the pixel density of the computed tomography scans. Equally important, the element distortion in some areas especially at the apical and cervical regions will result in inaccurate FE solution of the whole FE model analyses [18, 19]. In addition to that, canine root was not fully modeled in order to reduce model and FE mesh size and subsequently solution time. However, this undoubtedly has increased the limitations of this 3D-FE study and its clinical inferences.

The maximum von Mises stresses generated by both axial and lateral loading were located buccally at the enamel margins next to the CEJ. However the maximum values of these stresses were four times higher under lateral loading compared with axial loading (Figure 6). In either case, the maximum stresses exceeded the reported values of maximum tensile strength of enamel and dentine [20, 21]. Enamel has suffered much higher stresses than dentine especially at the cervical buccal CEJ region under lateral loading [22]. The enamel tooth tissue is known to be thin, having a very weak prismatic structure and low ultimate tensile strength at the CEJ. In addition, the CEJ is usually subject to erosion and abrasion, caused by acidic exposure and tooth brushing, respectively, which further weaken and undermine the structure of cervical enamel and dentine [23, 24]. Therefore, tensile stresses, along with other contributing factors, concentrated at the CEJ seem to be most attributable aetiology for abfraction lesions.

**Figure 7:**
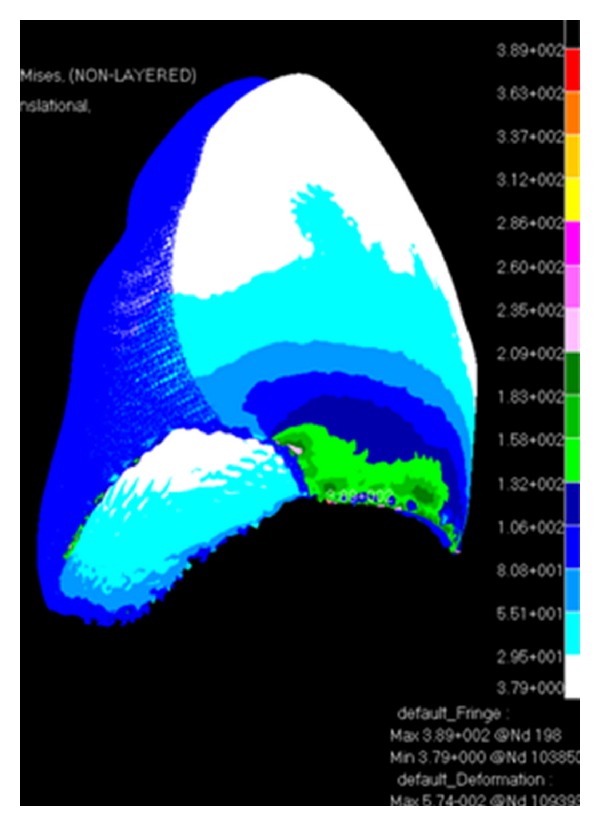
von Mises stress distribution (MPa) and strain (mm) of enamel tissues under lateral loading (100 N).

**Figure 8:**
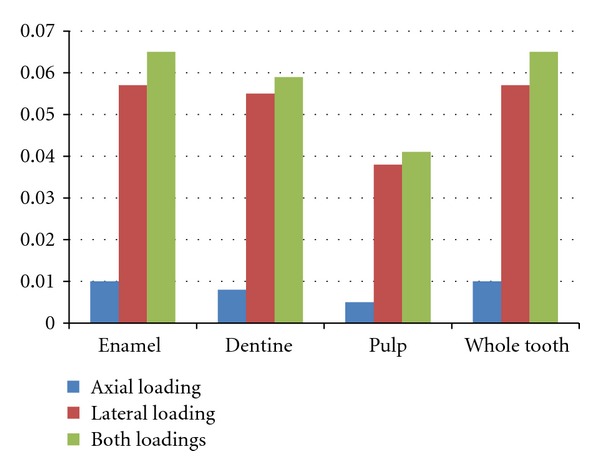
Strains (mm) of tooth tissues under different loading scenarios.

The non-axial loading presented in parafunctional activities (bruxism) is more detrimental than physiological occlusal loading in initiating cracks or fracture of the marginal buccal enamel at the CEJ (Figure 7). The maximum stresses caused by non-axial loading, which were concentrated at the buccal cervical region exceeded the maximum tensile strength of enamel as reported in the literature <17 MPa [21]. This provides further evidence for the role of bruxism in the formation of abfraction lesions. Enamel and dentine strains under different loading conditions were almost similar, however, the maximum strain values were observed at the buccal CEJ (Figure 8) which might have exacerbating consequence on the formation of abfraction lesion.

Albeit, some research findings have cast some doubt on abfraction as a prime cause for enamel and dentine breakdown at the buccal cervical region, the FE method has contributed to the prediction and biomechanical development of non-carious cervical lesions (abfraction) in upper canine teeth [24–26]. 3D-FEA has been validated by various experimental studies, and it was proved that micro-CT-based 3D-FEA is a valid research method [27, 28]. In this study 3D-FEA, the upper canine tooth model was reconstructed from a segmented micro-CT data which is a sophisticated model representing an upper canine tooth subjected to all possible occlusal loading scenarios to estimate the actual strains of enamel and dentine tissues within acceptable accuracy limits [29]. The fact that both axial and non-axial loading were investigated together in order to simulate parafunctional loading (Bruxism) which is usually pronounced with simultaneous loading in different direction. The findings of this study clearly indicated that this scenario has resulted in the worst biomechanical outcome with marked increase in stresses and displacement in the buccal cervical area especially within the enamel layer. This finding was also clinically proven that in bruxist patients are more prone to abfraction lesions than others [30], thereby; this group of patients needs close clinical monitoring and intervention when it is required.

## 6. Conclusions

Despite the limitations of 3D-FEA, the following conclusions might be drawn:

1. maximum stresses and crown displacement generated by lateral loading were generally higher than the vertical loading;
2. peak stresses were concentrated at the CEJ in all loading scenarios;
3. the greatest levels of stress generated within enamel and dentine were located at the CEJ when axial and non-axial loadings were applied simultaneously;
4. pulp tissues sustained the minimum level of stress under all loading conditions.

## Figures and Tables

**Table 1:** Mechanical properties of all materials included in the FE model.

Material	Young's modulus (MPa)	Poisson ratio
Enamel	84100	0.3
Dentine	18600	0.31
pulp	0.002	0.45

**Table 2:** Maximum von Mises stresses and strains sustained by tooth tissues under different occlusal loading scenarios.

Structure	Max. von Mises stress (MPa)			Strain (*μ*) of tooth tissues		
	Axial loading	Lateral loading	Both loadings	Axial loading	Lateral loading	Both loadings
Enamel	108	389	492	10	57	65
Dentine	73	293	342	8	55	59
Pulp	6.3	51.7	56.3	5	38	41
